# Using the missed opportunity tool as an application of the Lives Saved Tool (LiST) for intervention prioritization

**DOI:** 10.1186/s12889-017-4736-3

**Published:** 2017-11-07

**Authors:** Yvonne Tam, Luwei Pearson

**Affiliations:** 1 Institute for International Programs, Department of International Health, Bloomberg School of Public Health, Johns Hopkins University, 615 N. Wolfe Street, Baltimore, MD 21205 USA; 2 United Nations Children’s Fund UNICEF, 3 UN Plaza, New York City, NY10017 USA

## Abstract

**Background:**

The Missed Opportunity tool was developed as an application in the Lives Saved Tool (LiST) to allow users to quickly compare the relative impact of interventions. Global Financing Facility (GFF) investment cases have been identified as a potential application of the Missed Opportunity analyses in Democratic Republic of the Congo (DRC), Ethiopia, Kenya, and Tanzania, to use ‘lives saved’ as a normative factor to set priorities.

**Methods:**

The Missed Opportunity analysis draws on data and methods in LiST to project maternal, stillbirth, and child deaths averted based on changes in interventions’ coverage. Coverage of each individual intervention in LiST was automated to be scaled up from current coverage to 90% in the next year, to simulate a scenario where almost every mother and child receive proven interventions that they need. The main outcome of the Missed Opportunity analysis is deaths averted due to each intervention.

**Results:**

When reducing unmet need for contraception is included in the analysis, it ranks as the top missed opportunity across the four countries. When it is not included in the analysis, top interventions with the most total deaths averted are hospital-based interventions such as labor and delivery management in the CEmOC and BEmOC level, and full treatment and supportive care for premature babies, and for sepsis/pneumonia.

**Conclusions:**

The Missed Opportunity tool can be used to provide a quick, first look at missed opportunities in a country or geographic region, and help identify interventions for prioritization. While it is a useful advocate for evidence-based priority setting, decision makers need to consider other factors that influence decision making, and also discuss how to implement, deliver, and sustain programs to achieve high coverage.

**Electronic supplementary material:**

The online version of this article (10.1186/s12889-017-4736-3) contains supplementary material, which is available to authorized users.

## Background

Prioritizing health policies and programs in resource poor settings is particularly important to ensure scare resources are used effectively for the maximum return. There are various priority setting tools available to guide decision makers to assess potential mortality impact and cost effectiveness of health programs. The Lives Saved Tool is one among them that goes beyond considering potential impact and cost effectiveness of health interventions, but also account for context-specific effectiveness of interventions, has the ability to conduct prioritization in national and subnational levels, and the tool has been validated in African and South Asian settings [1–3].

### Missed Opportunity tool as a ‘first look’ at prioritizing interventions according to their impact

There have been published experiences of using LiST to guide decisions in country-level program planning in countries such as Burkina Faso, Ghana, Malawi, Ethiopia, and South Africa [4–6]. In order to provide an easier and quicker experience for users to identify and prioritize health interventions, an automated analysis called Missed Opportunity that assess impact of each proven interventions reaching every mother and child was recently added as a new tool in LiST. If there is no universal access to these interventions, the potential deaths averted will become “missed opportunities”.

### Global Financing Facility investment cases as an application of the Missed Opportunity tool

The Global Financing Facility (GFF) is a financing platform that garners financial support from multiple partners to fund reproductive, maternal, newborn, child, and adolescent health (RMNCAH) activities in low and middle income countries. There are country-led efforts to develop investment cases to state the prioritized activities the country wants to focus on to improve RMNCAH, and the associated funds needed. The investment cases are then used as a guide to mobilize resources from the national government and partner organizations [7]. As investment cases aim at prioritizing high impact, evidenced-based interventions, Missed Opportunity analyses were conducted to demonstrate utility of the tool for decision making. Missed opportunity results were used to compare the relative impact of interventions, and to prompt contextualized discussions to affect prioritization of intervention scale-up.

## Methods

The Spectrum software is a suite of policy models that is free and publicly available for use, and provides the necessary demography, HIV/AIDS, and family planning information for mortality impact modeling in LiST [8]. LiST projects mortality impact of women, stillbirths, and children based on changes in coverage of interventions that have a proven effect on reducing cause-specific mortality. Users can create projections, and utilize the default country-specific inputs, or specify their own to model the impact of about 70 maternal and child health interventions [9]. The Missed Opportunity tool, housed within the LiST module of the Spectrum software, automates the process of scaling up coverage of each of the 70 interventions individually to a target coverage at scale, and rank the interventions according to the magnitude of deaths averted by the interventions. Users can either use the default data or user-entered data to look at missed opportunities. The process in which the Missed Opportunity tool produces results is exactly the same as creating 70 projections in LiST for each country, and scale up one intervention in a projection at a time. Spectrum version 5.441 was used for this analysis to look at missed opportunities in pilot countries of GFF – Democratic Republic of the Congo (DRC), Ethiopia, Kenya, and Tanzania. Default model inputs such as baseline number of deaths, deaths by causes, mortality rates, and intervention coverage are included in Additional file 1.

### Missed Opportunity analyses using default national level data in LiST

The Missed Opportunity tool draws on national level data in LiST. The LiST database houses national intervention coverage estimates from large household surveys such as the Demographic Health Survey (DHS) or the Multiple Indicator Cluster Survey (MICS), and other national health status data – mortality rates, causes of death, disease incidences, and risk factors such as stunting and wasting prevalence. This database is updated as these new estimates become publicly available.

In the Missed Opportunity analysis using default data, coverage of each intervention in LiST is scaled up from its current coverage to 90% in the next year, while assuming coverage of all other interventions stay constant at its current coverage until the next year. The analysis can also be used to scale up contraceptive prevalence rate such that unmet need for contraception is reduced to 10%. 90% is chosen as the default target coverage at scale as it is an aspirational but achievable target, as evidenced by the coverage achieved by DPT3 vaccination in many low and middle income countries [10]. Any interventions with coverage at or above 90% will be left as is and not scaled down. The set of interventions assessed in a Missed Opportunity analysis varies for each country, depending on the level of current coverage of interventions and the burden of diseases. When users look at the missed opportunities of countries using default national level data from LiST, the analysis includes 86 countries with an under 5 mortality rate of 20 or higher in 2015 (Additional file 2).

### Missed Opportunity analyses using custom data

The Missed Opportunity tool is highly customizable for users who wish to use their own custom national or subnational level data, or for those who wish to set a coverage target other than 90%. Users will have to utilize the LiST model to create a projection using their custom data; subnational projections can be made quickly using the Subnational Projection tool in the LiST model. Users can then utilize the Missed Opportunity tool to create a Missed Opportunity file using their custom projection in order to visualize the intervention rankings according to additional deaths prevented.

Users may organize graphical results from the Missed Opportunity analysis according to the number of additional deaths prevented, by outcome type (maternal, stillbirths, or children), by delivery points of interventions, or according to causes of death. For step by step guidance on how to use the Missed Opportunity tool, please refer to the Spectrum help manual available for access via the software.

## Results

**Fig. 1:**
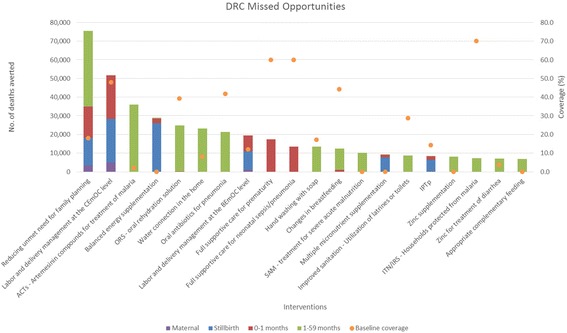
Total number of deaths averted per intervention in DRC*. *Only the top 20 interventions with the most deaths averted are shown

**Fig. 2:**
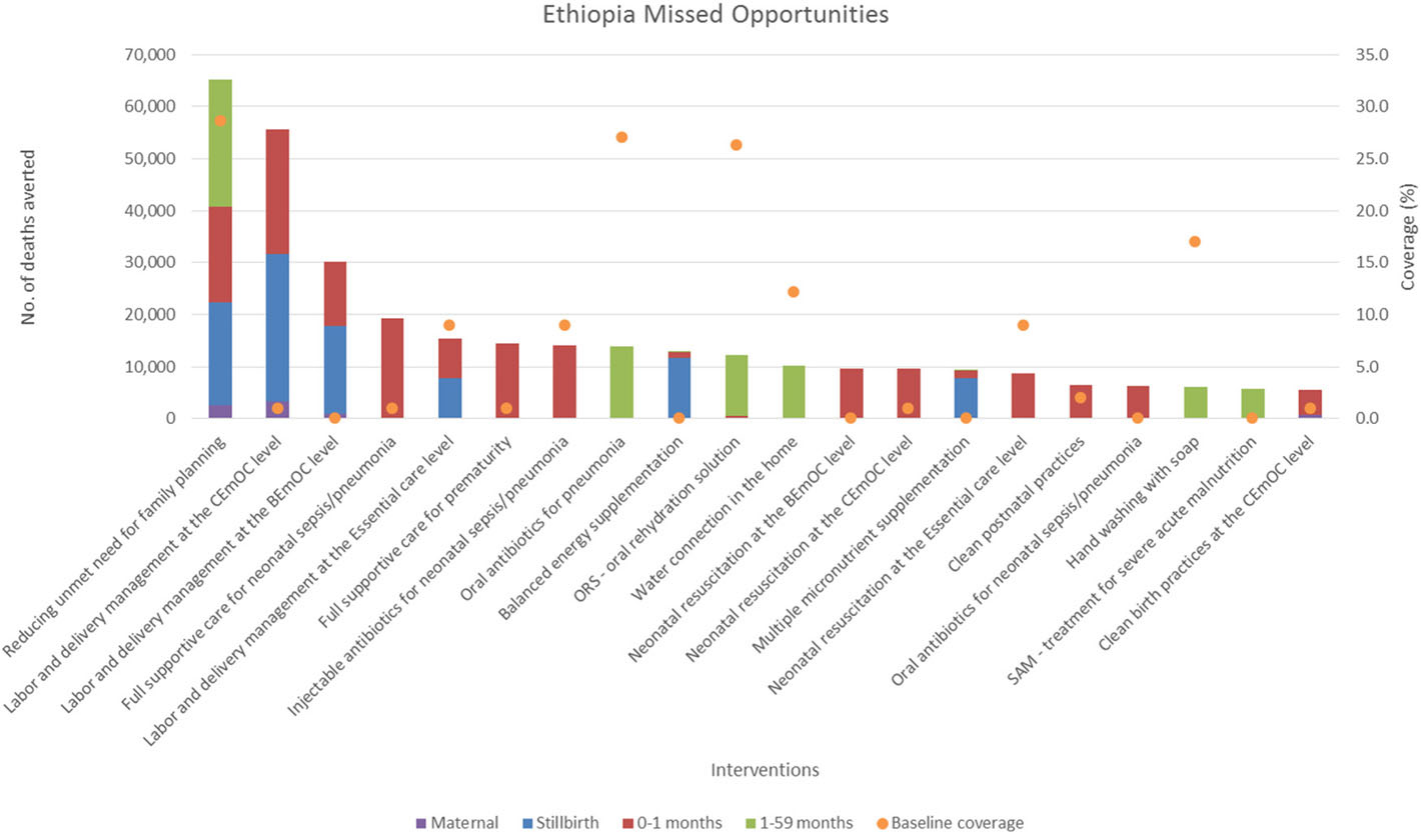
Total number of deaths averted per intervention in Ethiopia*. *Only the top 20 interventions with the most deaths averted are shown

**Fig. 3:**
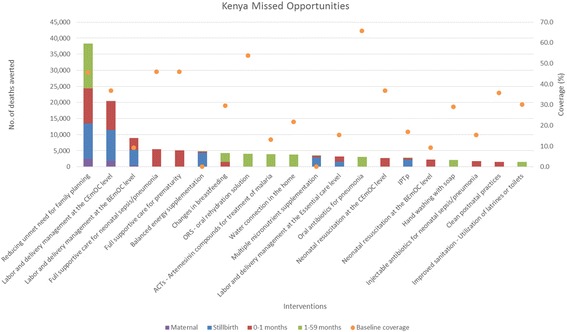
Total number of deaths averted per intervention in Kenya*. *Only the top 20 interventions with the most deaths averted are shown

**Fig. 4:**
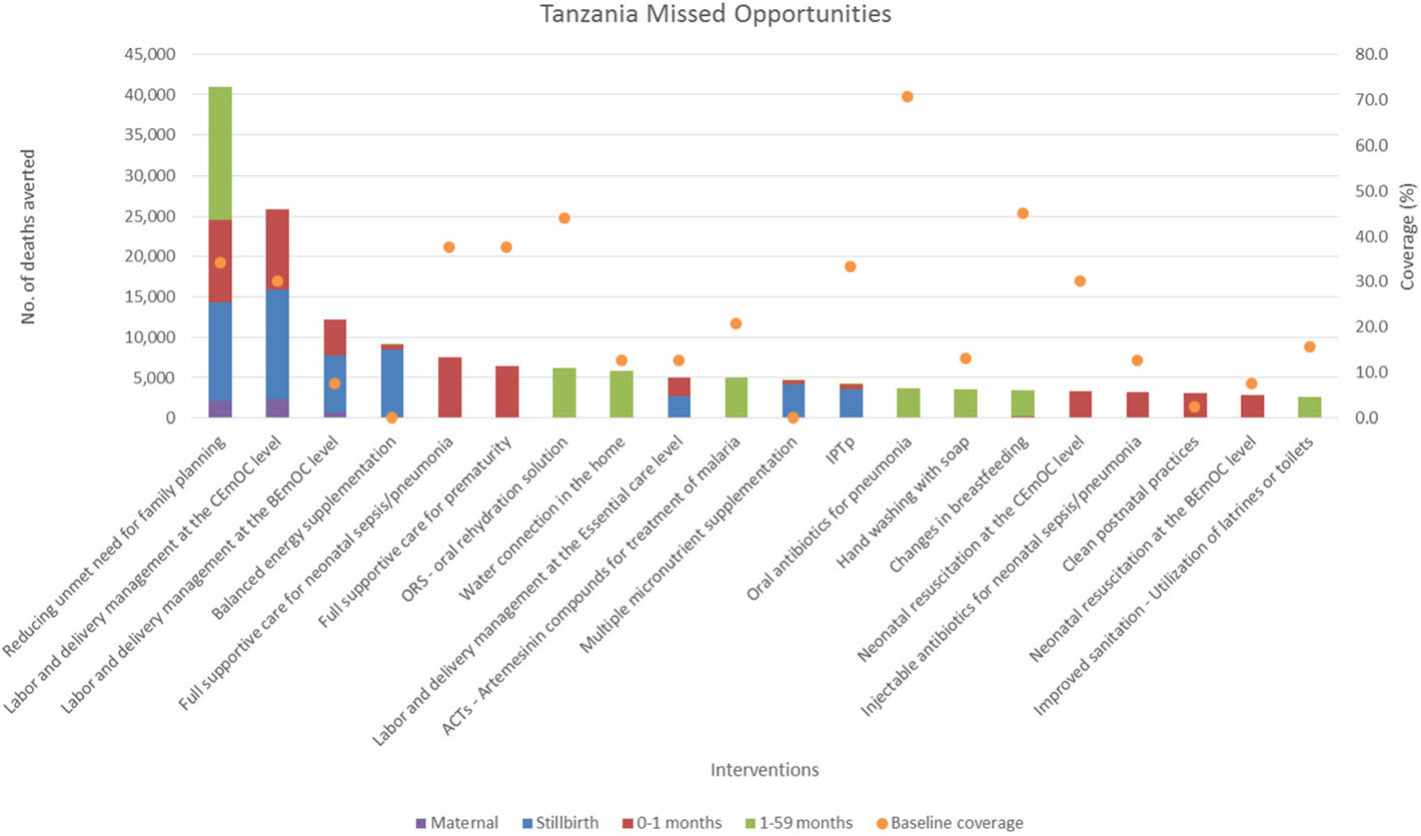
Total number of deaths averted per intervention in Tanzania*. *Only the top 20 interventions with the most deaths averted are shown

Missed opportunities for women, stillbirths, children less than 1 month of age, and children 1–59 months of age are shown by intervention in Figs. 1, 2, 3, and 4 for Democratic Republic of the Congo, Ethiopia, Kenya, and Tanzania. Detailed estimates of deaths averted by age groups are available in Additional file 1.

When reducing unmet need for family planning is included in the analysis, it averts the most total deaths for all age groups for all four countries. When reducing unmet need for family planning is not included, Table 1 shows the top five missed opportunities across all age groups. Labor and delivery management in the Comprehensive Emergency Obstetric Care (CEmOC) level, and the Basic Emergency Obstetric Care (BEmOC) level, full supportive care for neonatal sepsis/pneumonia, and for prematurity rank as the top interventions for averting total deaths. These are health facility-based interventions that are highly effective at saving lives [11–15]. Balanced energy supplementation for pregnant women is another top missed opportunity, as this proven intervention impact multiple death causes for stillbirths and children, but has not been rolled out in countries [16, 17]. Table 2 shows the top intervention by age groups that has averted the most deaths. Labor and delivery management in CEmOC level consistently averts the most maternal, stillbirths, and neonatal deaths in the four countries. The proportion of deliveries attended by skilled attendants at facilities are typically low in these countries, while receiving high quality care from a skilled attendant during labor and delivery at a CEmOC level facility is highly effective at averting deaths [11–13]. As for children 1–59 months, depending on the burden of disease and the baseline coverage of interventions, artemesinin – based combination therapies for treatment of malaria (ACTs), oral antibiotics for pneumonia, and oral rehydration solution for diarrhea (ORS) avert the most deaths for this age group. Definitions of interventions in Tables 1 and 2 can be found in Additional file 3.

**Table 1:** Top five missed opportunities in DRC, Ethiopia, Kenya, and Tanzania (1 - largest no. of total deaths averted)

	DRC^a^	Ethiopia^a^	Kenya^a^	Tanzania^a^
1	Labor and delivery management at the CEmOC level	Labor and delivery management at the CEmOC level	Labor and delivery management at the CEmOC level	Labor and delivery management at the CEmOC level
2	ACTs - Artemesinin –based combination therapies for treatment of malaria	Labor and delivery management at the BEmOC level	Labor and delivery management at the BEmOC level	Labor and delivery management at the BEmOC level
3	Balanced energy supplementation	Full supportive care for neonatal sepsis/pneumonia	Full supportive care for neonatal sepsis/pneumonia	Balanced energy supplementation
4	ORS - oral rehydration solution	Labor and delivery management at the Essential care level	Full supportive care for prematurity	Full supportive care for neonatal sepsis/pneumonia
5	Water connection in the home	Full supportive care for prematurity	Balanced energy supplementation	Full supportive care for prematurity

^a^If reducing unmet need for family planning is included in the Missed Opportunity analysis, it averts the most total deaths for all four countries

**Table 2:** Top interventions with most deaths averted by age groups in DRC, Ethiopia, Kenya, and Tanzania

	DRC	Ethiopia	Kenya	Tanzania
Maternal	Labor and delivery management at the CEmOC level	Labor and delivery management at the CEmOC level	Labor and delivery management at the CEmOC level^a^	Labor and delivery management at the CEmOC level
Stillbirth	Balanced Energy Supplementation	Labor and delivery management at the CEmOC level	Labor and delivery management at the CEmOC level^a^	Labor and delivery management at the CEmOC level
0-1 months	Labor and delivery management at the CEmOC level	Labor and delivery management at the CEmOC level	Labor and delivery management at the CEmOC level^a^	Labor and delivery management at the CEmOC level^a^
1-59 months	ACTs – Artemesinin-based combination therapies for treatment of malaria^a^	Oral antibiotics for pneumonia^a^	ORS - oral rehydration solution^a^	ORS - oral rehydration solution^a^

^a^If reducing unmet need for family planning is included in the Missed Opportunity analysis, it averts the most deaths for this age group

## Discussion

The Missed Opportunity tool is intended to provide a quick, first look for users who may or may not have an in depth knowledge of the current health status and intervention coverages of the countries of interest, or the relative efficacy of interventions. Users can produce missed opportunity results with a few clicks in the Spectrum software, which will make projecting mortality impact easily accessible for those without extensive prior knowledge on how to use Spectrum or LiST. The Missed Opportunity tool was set up as an automated analysis when the LiST team has received multiple requests from donors and bilaterals to conduct the same analyses – to quantify the impact of a hypothetical scenario of interventions reaching universal coverage. Since then, the Missed Opportunity tool has been used in ad hoc instances as a first look at impact, and few instances that were documented and published [18].

### Understanding results from Missed Opportunity analyses

Interventions with low current coverage that are highly effective at reducing main burden of diseases would emerge as the largest missed opportunities. As each intervention’s coverage scale-up was analyzed in isolation of other interventions, these projected deaths averted attributed to each intervention are the maximum possible impact of that intervention. Missed opportunities from different interventions cannot be summed together, as one will double count deaths averted from different interventions that are effective in reducing the same cause-specific deaths.

As seen from the four countries, facility-based interventions and treatment interventions are typically the most impactful at averting deaths, when reducing unmet need for family planning is not included in the Missed Opportunity analysis. Reducing unmet need for family planning increases contraceptive prevalence, and in turn changes the mix of contraceptive methods and reduces fertility. The deaths averted shown in the Missed Opportunity analysis for this intervention encompass two pathways. A very small proportion of the deaths averted are due to a reduction of risky births, which in turn improves birth outcomes and avert deaths. Majority of the deaths averted are due to a reduction in number of pregnancies and births [9]. Some may choose to not consider these as deaths averted, as these were children never born and therefore did not die. Users should interpret the relative impact of reducing unmet need for family planning compared to other interventions critically with this in mind.

### Using Missed opportunity results for intervention prioritization

Prioritization of interventions depends on a whole host of factors – political will, societal preferences, funding availability, amongst many other factors. The Missed Opportunity analysis provide decision makers the number of potential lives saved as the normative factor to prioritize high impact interventions that are evidence-based. Ethiopia and Tanzania have both identified scaling up interventions delivered in BEmOC and CEmOC level facilities as priorities in their national health plans as well as their GFF investment cases. Results from this analysis, which identified BEmOC and CEmOC level interventions as the top missed opportunities, can be used to strengthen the case for prioritizing these interventions. As Missed Opportunity analyses are rather quick and easy to generate, it lends itself to being used frequently to capture changes in lives saved should there be changes in other more upstream aspects of the health system that improves coverage. Missed opportunity results can be generated regularly to populate country profiles for initiatives such as the Countdown to 2030 to track changes in lives saved as health status in countries improve. Missed opportunity results can also be generated to help governments monitor changes to projected lives saved when there are changes to health indicators according to RMNCAH scorecards.

### Contextualizing Missed opportunity results to affect decision making

Missed opportunity results can also initiate discussions among key players in RMNCAH on how to efficiently implement, deliver, and sustain coverage of these interventions in an equitable manner at scale, such that they will achieve their projected impact. As the scale-up of these interventions heavily depend on health system readiness and require multi-sector collaboration, it is also important to involve non-health partners in discussion and planning. In Ethiopia for example, results from our Missed Opportunity analysis support the focuses of the Ministry of Health according to their Health Sector Development Program (HSDP) IV. HSDP has specific targets for scaling up skilled birth attendance and institutional delivery, upgrading health facilities to provide BEmOC and CEmOC level of care, and neonatal case management [19]. However, these interventions might be less feasible to implement at scale in short term, compared to community-based interventions, as they require more resources. Hospital-based interventions are also less likely to be equitable, especially when majority of the Ethiopian population live in rural areas with fewer high level health facilities than urban areas [5]. Missed opportunity results are intended for users to rapidly compare the relative impact of interventions, and will then require in-depth, contextualized discussions to affect priority setting. For example, a local policy dialogue is required to determine where and who should provide treatment and support to newborns with infection and children with pneumonia and diarrhea, as well as how to make the treatment affordable to the poorest households.

Although the Missed Opportunity analysis scales up coverage of intervention in isolation of others, it by no means encourage the implementation of individual vertical programs that emphasize a single intervention, versus delivering packages of preventive and treatment interventions to combat multiple diseases through a horizontal, primary health care system [20]. The case of synergy is evident – skilled birth attendant, emergency obstetric care and family planning are critical for the reduction of maternal and newborn deaths as well as stillbirth. Any Missed Opportunity analysis should not take away from a thoughtful local analysis – one that carefully assesses the input data, and has substantiated assumptions for scale-up that consider the capacities of local health systems. A LiST analysis can then offer valuable information to project the return for investment, as interventions costs can also be assessed using the costing tool in LiST that uses an ingredients-based approach to account for costs of drug and supply, labor, capital, and other recurrent costs [21].

### Limitations of the Missed Opportunity tool

It is also important to recognize the limitations of Missed Opportunity analyses. The Missed opportunities results currently only include deaths averted as an outcome, and will expand to provide stunting averted and non-severe cases of diseases averted in the future. Validity of the results depends on the quality of the input data for modeling. Although the four GFF priority countries all have recently published coverage surveys, coverage estimates from large household surveys are typically collected from a few years prior to publication. Assuming that coverage of interventions generally increases over time, using older estimates will overestimate the impact of intervention scale-up. It is also important to recognize that these coverage estimates were used under the assumption that the health interventions received were delivered with the best quality. Without an actual measurement of the quality of the health interventions delivered, one can assume that the coverage estimates from household surveys – one that measures only need and use without quality, are an over-estimate of the effective coverage, and hence underestimating the missed opportunities. Finally, the Missed Opportunity analysis is modeling the aspirational scenario of bringing coverage of all interventions to those who need it in one year. If coverage of skilled birth attendants and facility delivery, used as proxies for coverage of facility-based interventions during childbirth, increase according its historical coverage, universal coverage can be reached by year 2035 [22]. While lives saved are a quantifiable factor to justify prioritization, it does not suggest the necessary steps for a health system to achieve and sustain universal coverage of interventions. For example, promoting universal coverage of the top missed opportunity – labor and delivery management in the CEmOC level – will likely involve discussions such as equitable provision of CEmOC level facilities, transportation and referral to such facilities, and addressing economic and cultural barriers to access care [23]. Having these life-saving services available will also require complementary effort on quality improvement, community mobilization, financial protection and other measures to ensure utilization of these services.

## Conclusions

The Missed Opportunity tool can be used as an advocate for evidence-based priority setting. As the automated analysis allows users to quickly compare the relative impact of interventions, it is most useful for users without extensive knowledge about the current health status of the country, or prior knowledge on how to use Spectrum and LiST for mortality modeling. Decision makers should use results from the Missed Opportunity tool to generate local discussions on how to prioritize, implement, deliver, and sustain health programs, or to further utilize LiST to model impact of comprehensive packages of health programs according to the interventions prioritized using the Missed Opportunity tool.

## Additional files

Additional file 1: Missed opportunities and baseline status of DRC, Ethiopia, Kenya, and Tanzania. This file contains the full list of missed opportunities for DRC, Ethiopia, Kenya, and Tanzania. Note that only the top 20 missed opportunities per country were shown in figures in the manuscript. Baseline number of deaths, mortality rates, and causes of deaths are also available. (XLSX 138 kb)

Additional file 2: List of countries included in Missed Opportunity analysis. This file contains the full list of countries included in the Missed Opportunity analysis in the Lives Saved Tool (LiST). The analysis includes 86 countries with an under 5 mortality rate of 20 or higher in 2015. (XLSX 17 kb)

Additional file 3: List of interventions and their definitions. This file contains the definitions of interventions that correspond to the top 5 missed opportunities in DRC, Ethiopia, Kenya, and Tanzania. Also included are definitions of delivery levels for labor and delivery management. (DOCX 17 kb)

## Abbreviations

ACTs: Artemesinin-based combination therapies
BEmOC: Basic Emergency Obstetric Care
CEmOC: Comprehensive Emergency Obstetric Care
DHS: Demographic Health Survey
GFF: Global Financing Facility
HSDP: Health Sector Development Program
LiST: Lives Saved Tool
MICS: Multiple Indicator Cluster Survey
ORS: Oral rehydration solution for diarrhea
RMNCAH: Reproductive, maternal, newborn, child, and adolescent health

## References

[1] Rudan I, Kapiriri L, Tomlinson M, Balliet M, Cohen B, Chopra M. Evidence-Based Priority Setting for Health Care and Research: Tools to Support Policy in Maternal, Neonatal, and Child Health in Africa. PLoS Med. 2010;7(7): e1000308. https://doi.org/10.1371/journal.pmed.1000308. 10.1371/journal.pmed.1000308 PMC2903581 20644640

[2] Friberg IK, Bhutta ZA, Darmstadt GL, Bang A, Cousens S, Baqui AH (2010). Comparing modelled predictions of neonatal mortality impacts using LiST with observed results of community-based intervention trials in South Asia. Int J Epidemiol.

[3] Hazel E, Gilroy K, Friberg I, Black RE, Bryce J, Jones G (2010). Comparing modelled to measured mortality reductions: applying the Lives Saved Tool to evaluation data from the Accelerated Child Survival Programme in West Africa. Int J Epidemiol.

[4] Bryce J, Friberg IK, Kraushaar D, Nsona H, Afenyadu GY, Nare N (2010). LiST as a catalyst in program planning: experiences from Burkina Faso, Ghana and Malawi. Int J Epidemiol.

[5] Onarheim KH, Tessema S, Johansson KA, Eide KT, Miljeteig I, Norheim OF (2012). Prioritizing child health interventions in Ethiopia: modeling impact on child mortality, life expectancy and inequality in age at death. PLoS One.

[6] McGee SA, Chola L, Tugendhaft A, Mubaiwa V, Moran N, McKerrow N, et al. Strategic planning for saving the lives of mothers, newborns and children and preventing stillbirths in KwaZulu-Natal province South Africa: modelling using the Lives Saved Tool (LiST). BMC Public Health. 162015. 10.1186/s12889-015-2661-x PMC4719569 26786979

[7] Desalegn H, Solberg E, Kim JY (2015). The Global Financing Facility: country investments for every woman, adolescent, and child. Lancet.

[8] Stover J, McKinnon R, Winfrey B (2010). Spectrum: a model platform for linking maternal and child survival interventions with AIDS, family planning and demographic projections. Int J Epidemiol.

[9] Walker N, Tam Y, Friberg IK (2013). Overview of the Lives Saved Tool (LiST). BMC Public Health.

[10] WHO. WHO vaccine-preventable diseases: monitoring system 2016 global summary - Time Series: DTP3 2016. [Cited on 2017 March 29] Available from: http://apps.who.int/immunization_monitoring/globalsummary/timeseries/tscoveragedtp3.html.

[11] Lee AC, Cousens S, Darmstadt GL, Blencowe H, Pattinson R, Moran NF (2011). Care during labor and birth for the prevention of intrapartum-related neonatal deaths: a systematic review and Delphi estimation of mortality effect. BMC Public Health.

[12] Pollard SL, Mathai M, Walker N (2013). Estimating the impact of interventions on cause-specific maternal mortality: a Delphi approach. BMC Public Health.

[13] Yakoob MY, Ali MA, Ali MU, Imdad A, Lawn JE, Van Den Broek N (2011). The effect of providing skilled birth attendance and emergency obstetric care in preventing stillbirths. BMC Public Health.

[14] Bhutta ZA, Das JK, Bahl R, Lawn JE, Salam RA, Paul VK (2014). Can available interventions end preventable deaths in mothers, newborn babies, and stillbirths, and at what cost?. Lancet (London, England).

[15] Zaidi AK, Ganatra HA, Syed S, Cousens S, Lee AC, Black R (2011). Effect of case management on neonatal mortality due to sepsis and pneumonia. BMC Public Health.

[16] Imdad A, Bhutta ZA (2011). Effect of balanced protein energy supplementation during pregnancy on birth outcomes. BMC Public Health.

[17] Ota E, Hori H, Mori R, Tobe-Gai R, Farrar D (2015). Antenatal dietary education and supplementation to increase energy and protein intake. Cochrane Database Syst Rev.

[18] Tam Y, Huicho L, Huayanay-Espinoza CA, Restrepo-Méndez MC (2016). Remaining missed opportunities of child survival in Peru: modelling mortality impact of universal and equitable coverage of proven interventions. BMC Public Health.

[19] Federal Democratic Republic of Ethiopia. Ministry of Health. Health Sector Development Programme IV: 2010/11 – 2014/15. Federal Democratic Republic of Ethiopia Ministry of Health. 2010.

[20] Victora CG (2010). Commentary: LiST: using epidemiology to guide child survival policymaking and programming. Int J Epidemiol.

[21] Adesina A, Bollinger LA (2013). Estimating the cost-savings associated with bundling maternal and child health interventions: a proposed methodology. BMC Public Health.

[22] Amouzou A, Richard SA, Friberg IK, Bryce J, Baqui AH, El Arifeen S (2010). How well does LiST capture mortality by wealth quintile? A comparison of measured versus modelled mortality rates among children under-five in Bangladesh. Int J Epidemiol.

[23] Campbell OM, Calvert C, Testa A, Strehlow M, Benova L, Keyes E (2016). The scale, scope, coverage, and capability of childbirth care. Lancet.

