# Supplementary material for: Using the missed opportunity tool as an application of the Lives Saved Tool (LiST) for intervention prioritization
Source: BMC Public Health. 2017 Nov 7;17(Suppl 4):735. doi: 10.1186/s12889-017-4736-3 (PMC5688441; doi:10.1186/s12889-017-4736-3)
Supplement: Supplementary file 3 — List of interventions and their definitions. This file contains the definitions of interventions that correspond to the top 5 missed opportunities in DRC, Ethiopia, Kenya, and Tanzania. Also included are definitions of delivery levels for labor and delivery management. (DOCX 17 kb) [file 12889_2017_4736_MOESM3_ESM.docx]

| Interventions | Definitions |
| --- | --- |
| ACTs - Artemesinin compounds for treatment of malaria | Percent of children treated within 48 hours of the onset of fever in malaria-endemic areas with an artmesinin-containing compound (artemisinin-based combination therapy, or ACT). |
| Balanced energy supplementation | Percent of pregnant women who are food insecure who receive balanced energy-protein (BEP) supplementation. |
| Full supportive care for neonatal sepsis/pneumonia | Percent of neonates with suspected sepsis/pneumonia treated with hospital-based full supportive care, including oxygen, IV fluids, IV antibiotics, blood transfusion, phototherapy, etc. as needed. |
| Full supportive care for prematurity | Percent of prematurely born neonates who have access to and receive hospital-based full supportive care, including KMC, feeding support/IV fluids, infection prevention/management, oxygen provision, management of neonatal jaundice, nasal CPAP/IPPV (as required), and surfactant for Respiratory Distress Syndrome. |
| Labor and delivery management* | Percent of women receiving labor and delivery management from a skilled birth attendant. However, the nature of this labor and delivery management is assumed to differ based on the location at which the delivery occurs (because of the differing availability of supplies, equipment, and skills of birth attendants at different levels of care). For this reason, the intervention of “labor and delivery management” has different effectiveness values at different levels of care. |
| Oral antibiotics for pneumonia | Percent of prematurely born neonates who have access to and receive hospital-based full supportive care, including KMC, feeding support/IV fluids, infection prevention/management, oxygen provision, management of neonatal jaundice, nasal CPAP/IPPV (as required), and surfactant for Respiratory Distress Syndrome. |
| ORS - oral rehydration solution | Percent of children 0-59 months with suspected diarrhea treated with oral rehydration solution (ORS), including sachets or pre-mixed solutions. This indicator does not include homemade sugar-salt solution or recommended home fluids due to lack of adequate data. |
| Water connection in the home | Percent of households with a household connection, including water piped into the home or yard. |

Table 1. Definitions of the top five Missed Opportunities in DRC, Ethiopia, Kenya, and Tanzania.
*Definition of delivery levels of labor and delivery management in Table 2 below.

| *Delivery levels for labor and delivery management | Definitions |
| --- | --- |
| Essential care | Deliveries at this level of care are assumed to be in facilities that include monitoring of labor progress with a partograph, detection of complications, infection control via a clean delivery, and episiotomy if needed. For the neonate, this includes routine care practices including immediate drying, skin-to-skin contact or immediate wrapping for thermal care, and clean cord cutting. |
| Basic Emergency Obstetric and Newborn Care (BEmOC) | Deliveries at this level of care are assumed to be in facilities that meet the WHO’s guidelines for BEmOC. BEmOC facilities must be able to perform seven signal functions: administer parenteral antibiotics; administer parenteral anticonvulsants; administer parenteral oxtyocics; manual removal of placenta; removal of retained products (manual vacuum aspiration); assisted vaginal delivery (with vacuum extractor or forceps); and neonatal resuscitation with bag and mask. |
| Comprehensive Emergency Obstetric and Newborn Care (CEmOC) | Deliveries at this level of care are assumed to be in facilities that meet the WHO’s guidelines for CEmOC. CEmOC facilities must be able to perform the seven signal functions of BEmOC, plus surgery (e.g. Caesarean section) and blood transfusion. |

Table 2. Definitions of delivery levels of labor and delivery management
